# Quantitative analytical frameworks for benefit-risk assessment in health technology assessment

**DOI:** 10.1590/S2237-96222026v35e20251400.en

**Published:** 2026-06-24

**Authors:** Erica Aranha Suzumura, Aline Frossard Ribeiro Bortoluzzi, Ana Carolina Nonato, Fernando Henrique de Albuquerque Maia, Sidney Marcel Domingues, Bruna de Oliveira Ascef, Alessandro Gonçalves Campolina, Beate Jahn, Uwe Siebert, Patrícia Coelho de Soárez

**Affiliations:** 1 Universidade de São Paulo, Faculdade de Medicina, São Paulo, SP, Brazil Universidade de São Paulo Faculdade de Medicina São Paulo SP Brazil; 2 UMIT TIROL - University for Health Sciences and Technology, Institute of Public Health, Medical Decision Making and Health Technology Assessment, Hall in Tirol, Tyrol, Austria UMIT TIROL - University for Health Sciences and Technology Institute of Public Health Medical Decision Making and Health Technology Assessment Hall in Tirol Tyrol Austria; 3 Universidade de São Paulo, Instituto do Câncer do Estado de São Paulo, São Paulo, SP, Brazil Universidade de São Paulo Instituto do Câncer do Estado de São Paulo São Paulo SP Brazil; 4 ONCOTYROL - Center for Personalized Cancer Medicine, Division of Health Technology Assessment, Innsbruck, Tyrol, Austria ONCOTYROL - Center for Personalized Cancer Medicine Division of Health Technology Assessment Innsbruck Tyrol Austria; 5 Harvard T.H. Chan School of Public Health, Center for Health Decision Science and Departments of Epidemiology and Health Policy & Management, Boston, MA, United States Harvard T.H. Chan School of Public Health Center for Health Decision Science Departments of Epidemiology and Health Policy & Management Boston MA United States; 6 Harvard Medical School, Mass General Brigham Center for Health Technology Assessment, Boston, MA, United States Harvard Medical School Mass General Brigham Center for Health Technology Assessment Boston MA United States

Analytical frameworks for benefit-risk assessment are decision support structures, which can be subdivided as descriptive and quantitative ^1^. While descriptive analytical frameworks provide qualitative instructions, quantitative analytical frameworks employ analytical models to perform benefit-risk assessment; however, they do not consist of mathematical algorithms that result in automated decisions ^1^^),(^^2^. This article presents quantitative analytical frameworks for benefit-risk assessment in the context of health technology assessment.

## What is the importance of quantitative analytical frameworks?

Some healthcare decisions regarding benefit-risk balance can be made easily. In situations where a technology increases potential benefits and decreases risks compared to an alternative technology (or vice versa, the technology presents fewer benefits and more risks than the comparator), a formal quantitative benefit-risk assessment with summary metrics may not be necessary. 

When a new technology offers more benefits and more risks (or fewer benefits and fewer risks), there is a “trade-off” situation, and the benefit-risk balance must be formally assessed and judged. When stakeholder preferences influence this balance, further quantitative assessment using quantitative analytical frameworks can be advantageous, if not crucial, for decision-making ^3^. Analytical models such as those used in economic evaluations (e.g., cost-effectiveness analysis) can be adapted to assess benefits and risks instead of (or in addition to) costs. 

## What are the most commonly used quantitative analytical frameworks?

A recent scoping review, focused on mapping publications on benefit-risk assessment methods, identified 28 types of quantitative analytical frameworks in the 83 documents it analyzed ^4^. Although none of these frameworks were created specifically for the context of health technology assessment, most are considered applicable to benefit-risk assessment throughout the entire life cycle of health technologies ^4^. The three most cited quantitative analytical frameworks in the publications were multicriteria decision analysis, mentioned in 62.7% of the documents, followed by Markov models (21.7%) and decision trees (19.3%). It should be noted that some of the publications may have cited more than one framework ^4^. All three frameworks use analytical models that can be employed in both benefit-risk assessment and economic evaluations. The main difference is that benefit-risk assessment ignores health costs and focuses on benefit outcomes (effectiveness) and risks (harms), while economic assessments do not always quantify risks or harms.

## Decision tree

The decision tree is a model with a notoriously simple and familiar structure, used to simulate the consequences of alternative technologies ^5^^),(^^6^. It enables the cumulative benefits and risks or harms of each technology at the end of the established time horizon to be estimated ^7^^),(^^8^. In this model, all possible paths that individuals or patients can take for all compared strategies are explicitly detailed, along with the probabilities and health consequences associated with each final outcome. Figure 1 shows an example of a decision tree.

**Figure 1:**
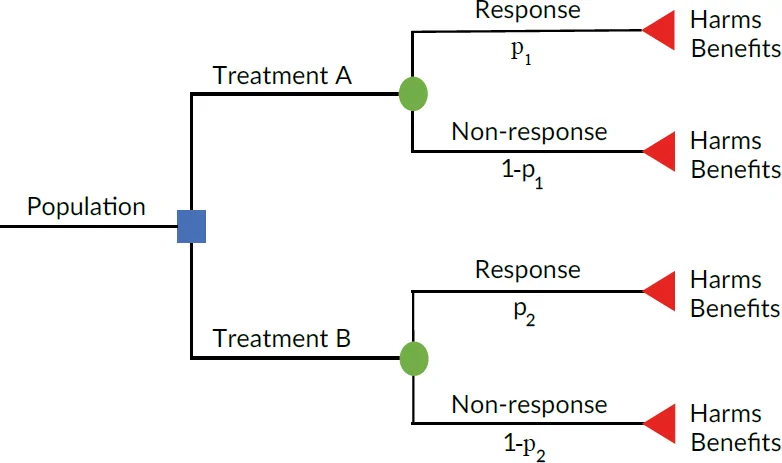
Example of a decision tree for comparing harms and benefits between treatments A and B, where “response” probabilities are represented by p1 and p2, and “non-response” probabilities are represented by their complements 1-p1 and 1-p2

In general, if the relevant time horizon is short, especially in the case of acute diseases, and mortality differs only over a short period, a simple decision tree is usually appropriate. However, its use is limited in situations in which it is necessary to assess a long time horizon or in situations in which repetition of events is possible ^9^.

## Markov model

Markov models are basically models that map the natural progression of a disease through multiple health states, as well as their transitions over time. There are two main types used in health decisions: Markov cohort models (or traditional models), which simulate cohorts over time, and state-transition models with individual simulation, known as microsimulation ^10^. Both cohort transition models and microsimulation models operate with Markov cycles, which are fixed time intervals during which transitions between health states occur. The duration of each cycle is defined by the modeler, adjusted to the simulated condition. In each cycle, individuals or fractions of the cohort transition to or remain in a given state according to specific probabilities. The total time horizon of the analysis is determined by the sum of these cycles ^11^.

As cohorts or individuals transition through health states over time, the health consequences associated with each state are computed. In benefit-risk assessment, the health consequences, called “rewards,” correspond to the benefits and the risks or harms ^7^.

Markov models are attractive for analyzing chronic diseases, since recurrence of events and explicit representation of time through cycles are easily incorporated ^5^. They are also interesting for longer time horizons and when the probabilities in the model vary over time ^10^. Figure 2 shows an example of a Markov model.

**Figure 2:**
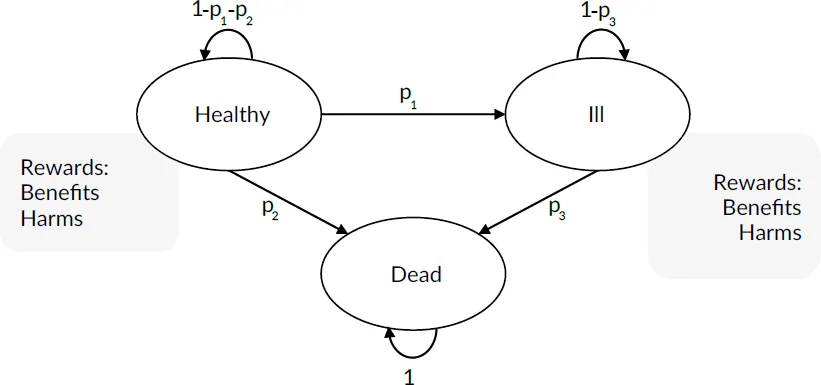
Example of a Markov model with three health states (healthy, ill and dead) for assessing benefits and harms, where the possibilities of transition between health states are represented by arrows, the probabilities of transition are represented by p1, p2 and p3, and the probabilities of remaining in the same state are represented by their complements (1, 1-p1-p2 and 1-p3)

## Multicriteria decision analysis

Multicriteria decision analysis is a set of methods and approaches for conducting a comparative analysis of several competing health technologies based on their performance against multiple and often conflicting criteria ^12^^),(^^13^. Methods for multicriteria decision analysis are classified into outranking models, reference-level models and value measurement models ^14^, the latter being the most widely used in health decisions ^15^.

Although there are differences in how these methods are applied, there are several elements of the process that are common to them: (1) defining the decision problem, (2) selecting and structuring criteria, (3) measuring the performance of alternatives, (4) scoring alternatives, (5) weighting criteria, (6) calculating aggregate scores, (7) dealing with uncertainty, and (8) interpreting and reporting findings ^14^. Figure 3 shows an example of multicriteria decision analysis. The International Society for Pharmacoeconomics and Outcomes Research (ISPOR) published a best practices report to support methodological planning and reporting of quantitative benefit-risk assessments based on multicriteria decision analysis ^16^.

**Figure 3:**
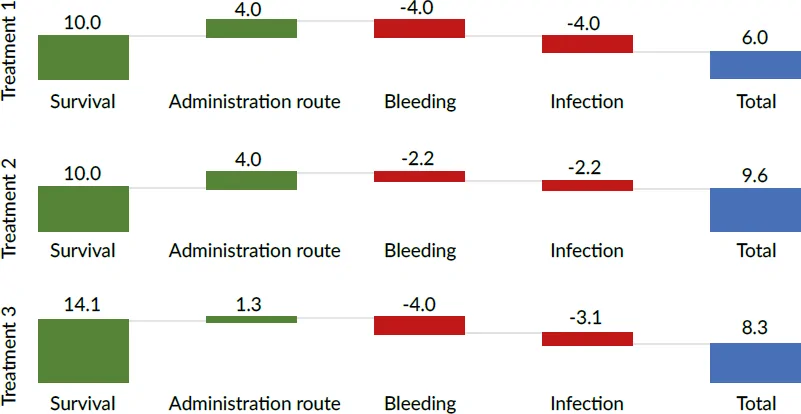
Example of multicriteria decision analysis of three treatment alternatives, with their respective scores for each criterion considered in the analysis (benefits shown in green, risks shown in red), and their aggregate scores (total shown in blue).

Multicriteria decision analysis offers the advantage of allowing the inclusion of patient and stakeholder preferences, as well as assessing a wider range of value dimensions, such as equity and innovation, often neglected in other analytical models ^15^^),(^^17^^),(^^18^. This analysis, however, presents significant challenges, such as lack of a homogeneous taxonomy ^1^^),(^^19^. In particular, there is an overlap between multicriteria decision analysis and some of the other models mentioned, such as decision trees and state-transition models.

## Selecting outcomes for benefit-risk assessment

Selection of outcomes for weighting in a benefit-risk assessment must be clear, plausible and predefined before the analysis is performed and should follow the same approach as selection of outcomes for descriptive benefit-risk assessment, as presented in the previous article in the series. It is crucial that clinical relevance be maintained; for example, it is not appropriate to use estimates of mild adverse events if data are available on serious and disabling events.

## Summary metrics

The quantitative results of the benefit-risk assessment can be presented using different summary or trade-off measures. Multicriteria decision analysis generally results in a total score for each alternative compared. Technologies with higher scores are preferred over others.

In Markov models or decision trees, some metrics are already known to readers familiar with economic assessments, such as quality-adjusted life years (QALYs) ^4^. Throughout the simulation, individuals may experience events characterized as benefits (such as avoided myocardial infarctions and cancers in remission) and harms (such as adverse events) depending on the probability of occurrence of each event. For each event, a respective utility score is assigned. At the end, the accumulated utility scores are multiplied by the respective resulting life years. If the difference in QALYs between the new technology and the comparator is positive (or negative), the new technology has a favorable (or unfavorable) benefit-risk balance. When comparing several alternatives, the alternative associated with a higher number of QALYs is considered to have the best benefit-risk balance.

Another metric is the so-called incremental harm-benefit ratio ^4^, which is analogous to the incremental cost-effectiveness ratio used in economic evaluations. It is calculated by dividing the incremental harms of alternative technologies by the incremental benefits. The incremental harm-benefit ratio is interpreted as the number of expected harms (events) for each additional unit of benefit achieved by a new technology compared to its alternative.

## Use of quantitative analytical frameworks in the context of health technology assessment in Brazil

Multicriteria decision analysis is not yet a common approach in deliberative processes for incorporating health technologies into the Brazilian Unified Health System (*Sistema Único de Saúde*), however, its use can be interesting for dealing with conflicting criteria and different stakeholder perspectives. On the other hand, models such as Markov models and decision trees are already widely used in complete economic evaluations (cost-effectiveness analysis and cost-utility analysis) ^20^, where they can be used to simultaneously assess multiple outcomes (benefits, harms, costs, equity etc.) and generate summary metrics such as the incremental cost-effectiveness ratio and the incremental harm-benefit ratio.

## Data Availability

The data are available within the body of the manuscript.
