# Supplementary material for: Quantitative analytical frameworks for benefit-risk assessment in health technology assessment
Source: Epidemiol Serv Saude. 2026 Jun 24;35:e20251400. doi: 10.1590/S2237-96222026v35e20251400.en (PMC13686422; doi:10.1590/S2237-96222026v35e20251400.en)
Supplement: Translation

# Translation: Estruturas analíticas quantitativas para avaliação benefício-risco na avaliação de tecnologias em saúde

**DOI:** 10.1590/S2237-96222026v35e20251400.pt

**Authors:** Erica Aranha Suzumura, Aline Frossard Ribeiro Bortoluzzi, Ana Carolina Nonato, Fernando Henrique de Albuquerque Maia, Sidney Marcel Domingues, Bruna de Oliveira Ascef, Alessandro Gonçalves Campolina, Beate Jahn, Uwe Siebert, Patrícia Coelho de Soárez

**Affiliations:** 1 Universidade de São Paulo, Faculdade de Medicina, São Paulo, SP, Brasil; 2 UMIT TIROL - University for Health Sciences and Technology, Institute of Public Health, Medical Decision Making and Health Technology Assessment, Hall in Tirol, Tirol, Áustria; 3 Universidade de São Paulo, Instituto do Câncer do Estado de São Paulo, São Paulo, SP, Brasil; 4 ONCOTYROL - Center for Personalized Cancer Medicine, Division of Health Technology Assessment, Innsbruck, Tirol, Áustria; 5 Harvard T.H. Chan School of Public Health, Center for Health Decision Science and Departments of Epidemiology and Health Policy & Management, Boston, MA, Estados Unidos; 6 Harvard Medical School, Mass General Brigham Center for Health Technology Assessment, Boston, MA, Estados Unidos

Estruturas analíticas (*frameworks*) para avaliação benefício-risco são estruturas de apoio à decisão, as quais podem ser subdivididas em descritivas e quantitativas ^1^. Enquanto as estruturas analíticas descritivas fornecem instruções qualitativas, as estruturas analíticas quantitativas empregam modelos analíticos para realizar a avaliação benefício-risco, contudo, elas não consistem em algoritmos matemáticos que resultam em decisões automatizadas ^1^^),(^^2^. Este artigo apresenta as estruturas analíticas quantitativas para avaliação benefício-risco no contexto da avaliação de tecnologias em saúde.

## Qual a importância das estruturas analíticas quantitativas?

Algumas decisões em saúde sobre o balanço benefício-risco podem ser tomadas facilmente. Em situações em que uma tecnologia aumenta os potenciais benefícios e diminui os riscos em comparação a outra tecnologia alternativa (ou vice-versa, a tecnologia apresenta menos benefícios e mais riscos do que o comparador), uma avaliação benefício-risco quantitativa formal com medida sumária pode não ser necessária. 

Quando uma nova tecnologia oferece mais benefícios e mais riscos (ou menos benefícios e menos riscos), há uma situação de “troca”, e o balanço benefício-risco deve ser formalmente avaliado e julgado. Quando as preferências das partes interessadas influenciam esse balanço, uma avaliação quantitativa adicional por meio de estruturas analíticas quantitativas pode ser vantajosa, se não crucial para a tomada de decisão ^3^. Modelos analíticos como os utilizados em avaliações econômicas (por exemplo, análise de custo-efetividade) podem ser adaptados para avaliar benefícios e riscos em vez de (ou adicionalmente aos) custos.

## Quais são as estruturas analíticas quantitativas mais usadas?

Uma recente revisão de escopo, focada em mapear publicações sobre métodos de avaliação benefício-risco, identificou 28 tipos de estruturas analíticas quantitativas em 83 documentos analisados ^4^. Embora nenhuma dessas estruturas tenha sido criada especificamente para o contexto da avaliação de tecnologias em saúde, a maioria é considerada aplicável à avaliação benefício-risco ao longo de todo o ciclo de vida das tecnologias em saúde ^4^. As três estruturas analíticas quantitativas mais citadas nas publicações foram a análise de decisão multicritérios, mencionada em 62,7% dos documentos, seguida pelos modelos de Markov (21,7%) e pelas árvores de decisão (19,3%), e uma publicação pode ter citado mais de uma estrutura ^4^. Todas as três estruturas utilizam modelos analíticos que podem ser empregados tanto na avaliação benefício-risco quanto na avaliação econômica. A principal diferença é que a avaliação benefício-risco ignora os custos em saúde e foca em desfechos de benefício (efetividade) e em riscos (danos), enquanto as avaliações econômicas nem sempre quantificam riscos ou danos. 

## Árvore de decisão

A árvore de decisão é um modelo com uma estrutura notoriamente simples e familiar, utilizada para simular as consequências de tecnologias alternativas ^5^^),(^^6^. Ela permite estimar os benefícios e os riscos ou danos acumulados de cada tecnologia ao final do horizonte temporal estabelecido ^7^^),(^^8^. Nesse modelo, todos os caminhos possíveis que os indivíduos ou os pacientes podem percorrer para todas as estratégias comparadas são explicitamente detalhados, juntamente com as probabilidades e as consequências em saúde associadas a cada desfecho final. A Figura 1 apresenta um exemplo de árvore de decisão.

**Figura 1:** Exemplo de árvore de decisão para comparação de danos e benefícios entre os tratamentos A e B, cujas probabilidades de “resposta” são representadas por p1 e p2, e de “não resposta” por meio de seus complementos 1-p1 e 1-p2

Em geral, se o horizonte temporal relevante for curto, especialmente em doenças agudas, e a mortalidade diferir apenas em um curto período, uma árvore de decisão simples é geralmente apropriada. Porém, seu uso é limitado em situações em que seja necessário avaliar um horizonte temporal longo ou em situações em que a repetição de eventos é possível ^9^.

## Modelo de Markov

Modelos de Markov são fundamentalmente modelos que mapeiam a progressão natural de uma doença através de múltiplos estados de saúde, bem como suas transições ao longo do tempo. Existem dois tipos principais utilizados em decisões em saúde: modelos de coorte de Markov (ou tradicionais), que simulam coortes ao longo do tempo, e modelos de transição de estados com simulação de indivíduos, conhecidos como microssimulação ^10^. Tanto os modelos com transição de coortes quanto os de microssimulação operam com ciclos de Markov, que são intervalos fixos de tempo durante os quais ocorrem as transições entre os estados de saúde. A duração de cada ciclo é definida pelo modelador, com ajuste à condição simulada. A cada ciclo, indivíduos ou frações da coorte transitam ou permanecem em um estado conforme probabilidades específicas. O horizonte temporal total da análise é determinado pela soma desses ciclos ^11^. 

Conforme coortes ou indivíduos transitam pelos estados de saúde ao longo do tempo, as consequências em saúde associadas a cada estado são computadas. Na avaliação benefício-risco, as consequências em saúde, chamadas de “recompensas”, correspondem aos benefícios e aos riscos ou danos ^7^.

Os modelos de Markov são atrativos para analisar doenças crônicas, visto que a recorrência de eventos e a explicitação do tempo por meio de ciclos são facilmente incorporadas ^5^. Também são interessantes para horizontes temporais maiores e para quando as probabilidades no modelo variam ao longo do tempo ^10^. A Figura 2 apresenta um exemplo de modelo de Markov.

**Figura 2:** Exemplo de modelo de Markov com três estados de saúde (saudável, doente e morto) para avaliação de benefícios e danos, cujas possibilidades de transição entre os estados de saúde são representadas por setas, as probabilidades de transição são representadas por p1, p2 e p3, e as probabilidades de permanência no mesmo estado representadas por seus complementos (1, 1-p1-p2 e 1-p3)

## Análise de decisão multicritérios

A análise de decisão multicritérios é um conjunto de métodos e de abordagens para realizar uma análise comparativa de várias tecnologias em saúde concorrentes com base em seu desempenho frente a critérios múltiplos e frequentemente conflitantes ^12^^),(^^13^. Os métodos para análise de decisão multicritérios se classificam em modelos de sobreclassificação, modelos de nível de referência e modelos de medição de valor ^14^, sendo este último o mais utilizado em decisões em saúde ^15^.

Embora existam diferenças nas formas em que esses métodos são aplicados, há vários elementos do processo que são comuns entre eles: (1) definir o problema de decisão, (2) selecionar e estruturar critérios, (3) medir o desempenho das alternativas, (4) pontuar alternativas, (5) ponderar critérios, (6) calcular pontuações agregadas, (7) lidar com a incerteza e (8) interpretar e relatar os achados ^14^. A Figura 3 apresenta um exemplo de análise de decisão multicritérios. A International Society for Pharmacoeconomics and Outcomes Research (ISPOR) publicou um relatório de boas práticas para apoiar o planejamento metodológico e o relato de avaliação benefício-risco quantitativas baseadas em análise de decisão multicritérios ^16^.

**Figura 3:** Exemplo de análise de decisão multicritérios entre três alternativas de tratamento, com suas respectivas pontuações para cada critério considerado na análise (benefícios apresentados em verde, riscos apresentados em vermelho), e suas pontuações agregadas (total apresentado em azul)

A análise de decisão multicritérios oferece a vantagem de permitir a inclusão de preferências de pacientes e de partes interessadas, além de avaliar uma gama mais ampla de dimensões de valor, como equidade e inovação, frequentemente negligenciadas em outros modelos analíticos ^15^^),(^^17^^),(^^18^. Essa análise, contudo, apresenta desafios significativos, como a falta de uma taxonomia homogênea ^1^^),(^^19^. Em especial, há uma sobreposição entre a análise de decisão multicritérios e alguns dos outros modelos mencionados, como árvores de decisão e modelos de transição de estado. 

## Seleção dos desfechos para a avaliação benefício-risco

A seleção dos desfechos para a ponderação em uma avaliação benefício-risco deve ser clara, plausível e predefinida antes da análise e deve seguir a mesma abordagem para a seleção dos desfechos para a avaliação benefício-risco descritiva, conforme apresentado no artigo anterior da série. É crucial que a relevância clínica seja mantida; por exemplo, não é apropriado usar estimativas de eventos adversos leves se houver dados disponíveis sobre eventos graves e incapacitantes.

## Medidas sumárias

Os resultados quantitativos da avaliação benefício-risco podem ser apresentados com uso de diferentes medidas sumárias ou de compensação. A análise de decisão multicritérios geralmente resulta em um escore total para cada alternativa comparada. Tecnologias com pontuações mais altas são preferíveis em relação às demais.

Nos modelos de Markov ou nas árvores de decisão, algumas medidas já são conhecidas pelos leitores familiarizados com avaliações econômicas, como os anos de vida ajustados pela qualidade (AVAQs) ^4^. Ao longo da simulação, indivíduos podem sofrer eventos caracterizados como benefícios (como infartos evitados e cânceres em remissão) e danos (como eventos adversos) a depender da probabilidade de ocorrência de cada evento. Para cada evento, um respectivo escore de utilidade é atribuído. Ao final, os escores de utilidade acumulados são multiplicados pelos respectivos anos de vida resultantes. Se a diferença de AVAQs entre a nova tecnologia e o comparador for positiva (ou negativa), a nova tecnologia tem um balanço benefício-risco favorável (ou não favorável). Na comparação de várias alternativas, aquela associada a um maior número de AVAQs é considerada a de melhor balanço benefício-risco.

Outra medida é a chamada de razão de dano-benefício incremental ^4^, que é análoga à razão de custo-efetividade incremental utilizada em avaliações econômicas. Ela é calculada pela divisão dos danos incrementais das tecnologias alternativas pelos benefícios incrementais. A razão de dano-benefício incremental é interpretada como o número de danos esperados (eventos) para cada unidade adicional de benefício alcançada pela nova tecnologia em comparação com a sua alternativa.

## Uso de estruturas analíticas quantitativas no contexto da avaliação de tecnologias em saúde no Brasil

A análise de decisão multicritérios ainda não é uma abordagem comum nos processos deliberativos para incorporação de tecnologias de saúde no Sistema Único de Saúde, porém, a sua utilização pode ser interessante para lidar com critérios conflitantes e com perspectivas das diferentes partes interessadas. Por outro lado, modelos como os de Markov e as árvores de decisão já são amplamente utilizados em avaliações econômicas completas (análise de custo-efetividade e análise de custo-utilidade) ^20^, em que podem ser aproveitados para avaliar simultaneamente múltiplos desfechos (benefícios, danos, custos, equidade, etc.) e gerar medidas sumárias como a razão de custo-efetividade incremental e a razão de dano-benefício incremental.

## Data Availability

Os dados estão disponíveis no corpo do manuscrito.
